# Dark feet and dark wings: penetrating the depths of the Earth

**DOI:** 10.1111/1468-5922.12868

**Published:** 2022-11-28

**Authors:** Robin B. Zeiger

**Affiliations:** ^1^ Tel Aviv Israel

**Keywords:** environmental destruction, dreams, collective unconscious, Jewish myth, ‘borderland’ personality, sandplay, destruction de l’environnement, rêves, inconscient collectif, mythe Juif, personnalité ‘frontière’, thérapie par le jeu de sable

## Abstract

The author, although an analyst, is an initiate into the topic of environmental destruction. Following Wendell Berry, she enters the dark and begins a journey of dream‐like reflection, weaving images from her own dream and drawing on the work of Vaughan, Bernstein, Soloveitchik and Sacks. She asks, ‘not if but where does climate change enter the room?’. The second half of the paper focuses on the manifestations of environmental destruction in dreams and sandplay from three patients and one dream group participant. The paper argues that the analyst must see and intuit before our patients can access the objective layer of environmental destruction in dreams and symbolic material. In this way, the climate becomes the wounded patient, and the analyst as wounded healer must first access his/her own relationship to the wounds inside. Finally, using an ancient Jewish mythological story of Rabbi Shimon bar Yochai, the author argues that Jungian analysts must work to find balance between the inner world of depth psychology and the outer world with its challenges and problems that include environmental destruction.

## Abstract (fr)

L’auteur, bien qu’étant analyste, est une initiée du sujet de la destruction de l’environnement. Inspirée par Wendell Berry, elle entre dans le noir et commence un voyage de réflexion en tissant des images provenant de son propre rêve et utilisant les travaux de Vaughan, Bernstein, Soloveitchik et Sacks. Pour elle la question est non pas si mais où est‐ce que le changement climatique entre dans la pièce ? La deuxième moitié de l’article s’intéresse aux manifestations de destruction de l’environnement dans les rêves et la thérapie par le jeu de sable de trois patients et d’une personne participant à un groupe de rêves. L’article soutient que l’analyste doit voir et suivre son intuition avant que les patients aient accès à la couche objective de la destruction de l’environnement dans les rêves et le matériel symbolique. De cette manière, le climat devient le patient blessé, et l’analyste, en tant que guérisseur blessé, doit tout d’abord accéder à sa propre relation aux blessures intérieures. Enfin, s’appuyant sur une ancienne histoire mythologique Juive, celle de Rabbi Shimon bar Yochai ‐ qui se retira dans une caverne pendant 13 années ‐ l’auteur soutient que les analystes Jungiens doivent œuvrer à trouver l’équilibre entre le monde intérieur de la psychologie des profondeurs et le monde extérieur avec ses défis et ses problèmes, qui incluent la destruction de l’environnement.

## Abstract (de)

Die Autorin, zugleich Analytikerin, ist eine Eingeweihte das Thema Umweltzerstörung betreffend. Wendell Berry folgend betritt sie die Dunkelheit und beginnt eine Reise der Reflexion indem sie Bilder aus ihrem eigenen Traum webt und sich auf die Arbeit von Vaughan, Bernstein, Soloveitchik und Sacks stützt. Sie fragt: 'Nicht ob, sondern wo kommt der Klimawandel in den Raum?' Die zweite Hälfte der Arbeit konzentriert sich auf Manifestationen der Umweltzerstörung in Träumen und Sandspielen von drei Patienten und einem Traumgruppenteilnehmer. Im Text wird argumentiert, daß der Analytiker sehen und intuitiv sein muß, bevor unsere Patienten auf die objektive Schicht der Umweltzerstörung in Träumen und symbolischem Material zugreifen können. Auf diese Weise wird das Klima zum verwundeten Patienten und der Analytiker, als verwundeter Heiler, muß zuerst auf seine eigene Beziehung zu den Wunden im Inneren zugreifen. Schließlich wird, unter Verwendung einer alten jüdischen mythologischen Geschichte von Rabbi Shimon bar Yochai, der sich 13 Jahre lang in eine Höhle zurückzog, argumentiert, daß Jungianische Analytiker daran arbeiten müssen, ein Gleichgewicht zwischen der inneren Welt der Tiefenpsychologie und der äußeren Welt mit ihren Herausforderungen und Problemen zu finden, was die Umweltzerstörung einschließt.

## Abstract (it)

L'Autore, sebbene analista, è un novizio al tema della distruzione ambientale. Seguendo Wendell Berry, entra nell'oscurità e inizia un viaggio di riflessione tessendo immagini dal proprio sogno e attingendo al lavoro di Vaughan, Bernstein, Soloveitchik e Sacks. Si chiede: "non se, ma dove entra nella stanza il cambiamento climatico?" La seconda metà del documento si concentra sulle manifestazioni di distruzione ambientale nei sogni e nel gioco della sabbia di tre pazienti e un partecipante al gruppo dei sogni. L'articolo sostiene che l'analista deve vedere e intuire prima che i nostri pazienti possano accedere allo strato oggettivo di distruzione ambientale nei sogni e nel materiale simbolico. In questo modo il clima diventa il paziente ferito, e l'analista come guaritore ferito deve prima accedere alla propria relazione con le ferite interne. Infine, utilizzando un'antica storia mitologica ebraica del rabbino Shimon bar Yochai, che si ritirò in una grotta per 13 anni, sostiene che gli analisti junghiani devono lavorare per trovare l'equilibrio tra il mondo interiore della psicologia del profondo e il mondo esterno con le sue sfide e problemi che includono la distruzione ambientale.

## Abstract (ru)

Хотя автор является аналитиком, она озабочена проблемой разрушения окружающей среды. Вслед за Уэнделлом Берри она вступает в темноту и начинает путешествие размышлений, переплетая образы своего собственного сновидения и данные работ Вогана, Бернстайна, Соловейчика и Сакса. Она спрашивает: "Не если, а где изменение климата входит к нам?" Вторая половина статьи посвящена проявлениям разрушения окружающей среды в сновидениях и песочных композициях трех пациентов и одного участника сновидческой группы. В статье утверждается, что аналитик должен обладать видением и интуицией, чтобы наши пациенты могли получить доступ к объективному слою разрушения окружающей среды в своих сновидениях и символическом материале. Таким образом, климат становится раненым пациентом, а аналитик, будучи раненым целителем, должен вначале получить доступ к своим собственным отношениям с внутренними ранами. Наконец, используя древний иудейский миф о рабби Шимоне бар Йохае, который уединился в пещере на 13 лет, она утверждает, что юнгианские аналитики должны работать над тем, чтобы найти баланс между внутренним миром глубинной психологии и внешним миром с его вызовами и проблемами, которые включают разрушение окружающей среды.

## Abstract (es)

La autora, es analista, y también iniciada en el tópico de la destrucción medioambiental. Siguiendo a Wendell Berry, ella entra en la oscuridad y comienza una travesía reflexiva hilvanando imágenes de sus propios sueños y apoyándose en los trabajos de Vaughan, Bernstein, Soloveitchik y Sacks. Ella pregunta, ‘¿sino dónde el cambio climático entra en la sala? La segunda parte del trabajo se focaliza en manifestaciones de la destrucción del medioambiente en sueños y en la caja de arena de tres pacientes y de un participante de un grupo de sueños. El trabajo argumenta que el/la analista debe poder ver e intuir antes que nuestros pacientes puedan acceder la dimensión objetiva de la destrucción medioambiental en sueños y material simbólico. De este modo, el clima deviene el/la paciente herido, y el/la analista como curador herido debe primero poder acceder su propia relación con su herida interior. Finalmente, utilizando una antigua historia mitológica de la tradición Judía del Rabino Shimon bar Yochai, quien se retiró a una cueva durante trece años, ella argumenta que analistas Junguianos deben trabajar para encontrar un balance entre el mundo interno de la psicología profunda y el mundo externo con sus desafíos y problemas como el de la destrucción ambiental.

## Abstract (zh)

黑脚和黑翼:穿透地球的深处。

作者虽然是一位分析师, 但却是环境破坏这一主题的发起者。继温德尔‐贝里之后, 她进入了黑暗, 开始了反思之旅, 从她自己的梦境中编织图像, 并借鉴了沃恩、伯恩斯坦、索洛维契克和萨克斯的作品。她问道:"不是如果, 而是气候变化在哪里进入房间?"论文的后半部分集中于三个病人和一个梦境小组参与者的梦境和沙盘游戏中环境破坏的表现。本文认为, 在我们的病人能够进入梦境和象征性材料中的环境破坏的客观层之前, 分析师必须看到和直觉到这部分。这样一来, 气候变成了受伤的病人, 而作为受伤的治疗者的分析师必须首先与他/她自己的内部伤口建立联系。最后, 她讲了一个古老的犹太神话故事, 这是一个关于拉比西蒙约凯在一个山洞里隐居了13年的故事, 借用这个故事, 她提出, 荣格分析师必须努力在深度心理学的内在世界, 和处于挑战和困难, 包括环境破坏的外部世界之间找到平衡。

To go in the dark with a light is to know the light. To know the dark, go dark. Go without sight,

and find that the dark, too, blooms and sings, and is travelled by dark feet and dark wings

Wendell Berry (2014)

## Introduction

In the words of Wendell Berry, sometimes we must dare to enter the dark and grapple with the strangeness found in the inner spaces of our Earth. In this dark place within *anima mundi*, is the possibility of a transformational new perspective. The call for papers for this special issue beckoned to me. With humility and curiosity, I began a journey in the dark that continues to enrich and transform my work as an analyst and teacher, and to touch my personal life. The journey began with a simple question, in my thirty plus years of clinical work, how often has climate change directly entered the temenos of my work? I informally surveyed several colleagues on two continents and the answer from most of us was a resounding, ‘almost never’. Were we all missing something? As Jungians, we believe in the archetypal layers to our soul and the objectivity of the collective unconscious. I knew I must ask *not if*, but *where* does climate change enter the room?

As analysts and as wounded healers, we accept the necessity of first touching our own internal wounded patient before we can escort others on their healing journey. I felt compelled to extend this idea and treat the environment as the patient. In retrospective reflection, I needed to meet the wounded environment inside, via the personal and collective unconscious. Only then could I meet the whisperings of these wounds in the consulting room and in the outer temenos of our deeply wounded *anima mundi*. The first half of my paper speaks of this journey. In the second half, I offer examples of my new process thinking about patients’ dreams and symbolic material through the lens of environmental destruction. Hillman speaks about the ‘multiplicity of meanings in dreams’ (1979, p. 126) and how they ‘restore to consciousness this sense of multiplicity’ (ibid., p. 41*)*. It is my thesis that environmental destruction does indeed sometimes enter my consulting room but sometimes in a whisper, via the many layers of dreams and other symbolic work.

## The unbound hero

Vaughan (2020) meets climate change with an emphasis on the hubris and ethnocentrism of the Western world and notes the chaos born out of the global climate crisis and the danger of over‐identification with the Western inflated ego.

It is in the news in many forms: fire, flood, extinction, refugees, rebellion. It has also entered the consulting room: clients are increasingly naming their fear, their despair, their rage, and their feelings of impotence in the face of the unknown.

(Vaughan 2020, pp. 48‐49)

We see such heroic narratives in our widespread tendency to externalize climate change as an enemy to be fought, something impinging on us from outside. These narratives cast us as innocent victims to whom climate change is happening. … But this crisis is not one that can be saved by heroically killing a monster. That archetype will not help us now. *We* are the monster.

(Vaughan 2020, p. 52, italics in original)

Bernstein (2005) also warns against the inflation and one‐sidedness of a Western ego that is over‐specialized and split off from nature: ‘the western ego construct is the organ of rationality. The exclusion of transrational reality from consideration leaves it unchecked by any power outside itself and prone to profound and dangerous inflation’ (p. 17).

Reflecting on the very different orientation of Navajo tribes to the world that is both deeply connected to nature and includes transrational reality, he writes:

By transrational reality I mean objective nonpersonal, nonrational phenomena occurring in the natural universe, information and experience that does not readily fit into standard cause and effect logical structure. These are the kinds of experience that typically are labeled and dismissed as superstition, irrational, and, in the extreme, abnormal or crazy.

(Bernstein 2005, pp. 16‐17)

Bernstein continues:

An ego cut off from a relationship with nature tends to be left with its own reflections on itself, unmediated by the transpersonal dimension, and readily trapped in its own mentalisms. Thus it is more prone to power inflations. It tends to be addicted to power and materialism, and thus has also spawned modern warfare with the capacity to eliminate life as we know it.

(ibid., pp. 33‐34)

In meeting Vaughan and Bernstein, I meet the environment as another patient in analysis and must begin to relate to the danger of this patient committing suicide.

## The lizards vs. the scientist: a personal dream

While thinking about this paper, I was reminded of a dream from several years ago:

In my mind’s eye, I see a little hole in the sand. There is a lizard’s head near the hole. I wanted to make sure he was alive. I thought I saw him breathing and tried to make him move. I see a lizard in a glass container with sand. My son‐in‐law, ‘Boomie’, wanted to take the lizards to work. He must have captured ‘my lizard’ to put him in the container with the second lizard. I was afraid one might kill the other.

A bit less than a decade prior to the dream, I had immigrated from city life in the United States to settle in a small Israeli agricultural village. I left a life of changing leaves, occasional snow and busyness to live in quiet. At the time, I didn’t understand the dream. Perhaps in consort with Bollas’ (1987) unthought known, the dream needed to incubate. Hillman reminds us, ‘the golden rule in touching any dream is keeping it alive’ (Hillman 1979, p. 116). Von Franz recollects, ‘Jung did not interpret his dreams by immediately forming a clear idea of what they meant; instead, he carried them around within himself, lived with them inwardly, as it were, and asked questions about them’ (von Franz 1998/1985, p. 23).

## The hole in the ground

The lizard was the opening character in this theatre of the soul, and I was frightened that he was dead (in my dream I ‘knew’ the lizards were male). Thankfully, he was very much alive. He rested next to a hole in the ground, facing the Earth with his phallic shaped body and the potential to penetrate the inner sanctuary. Pregnant with possibility, the scene reminded me of Mother Nature that births, nurtures, swallows, and destroys. It seemed likely the lizard knew how to negotiate this hole, yet my dream soul was concerned. The unsuspecting lizard was then plucked from free existence and placed inside a container presumably for the advancement of science. In consort with the theme of climate change and the environment, my dream soul was alarmed at humankind’s penetration of the natural order.

The archetypal pure scientist dissects, differentiates, and manipulates. The researcher did not seem to consider the implications of putting the lizards together in a small space and the observer‐me feared that one would kill the other. I am reminded of Bernstein’s (2005, pp. 8‐9) concern about the danger of the split between mind and nature in the over‐specialized Western ego. Dreams sometimes guide the way toward integration by way of the transcendent function. Perhaps the two lizards and the freedom vs. confinement theme both hinted at the struggle to integrate. Perhaps my dream arose from a liminal space: the place where our ‘heroic’ consciousness meets and struggles with the objective level of the collective unconscious, the great and terrible mother.

In my associations, Boomie hinted at a piece of myself within the larger world. A father’s daughter, I ‘grew up’ in graduate school when feminism was on the rise. My clinical programme was academic without enough space for the feminine and the soulful. Female professors were encouraged to embrace the patriarchal values of the time. In waking life, Boomie studies desert algae and he and my daughter work hard to live in harmony with the desert as much as possible, gardening, composting, recycling, and raising children to care and respect our world and its inhabitants. Unlike my previous struggles with the father complex, in this dream, my internal ‘Boomie’ represents a new masculine figure, the archetypal Man/Child of the Earth, who seems to be an internal ally in my/our fight for climate protection.

## The archetypal desert

Why is the setting of the dream the desert? As a Jew and an immigrant from America to Israel, I relate to the ‘wandering Jew’ of the desert. From time immemorial, humankind has been drawn to the desert as a powerful and archetypal space. Blessed and cursed with extremes, it is a place to be cherished and feared. The desert inspires religious figures, musicians, and artists. Georgia O’Keeffe found solace and inspiration from the sun‐bleached desert bones that she called her ‘symbols of the desert’. Pounders states:

Apparently for O’Keeffe, the bones represented both the allure and beauty of the wide‐open liberating spaces of the New Mexican desert as well as the contrasting sense of desolation and wildness. … I proposed that another way to view the bones is as an embodiment of O’Keeffe’s *prima materia* – objects that assumed the projection of her inner state.

(Pounders 2019, p. 20)

Pounders’ last sentence resonates with me. When I dreamt the dream years ago, I was puzzled. These lizards remained in the recesses of my consciousness to bother me. I now see the dream as *prima materia*, a reflection of an inner state that needed to incubate. In retrospect, this personal dream seemed to serve as psychopomp and deepened my connection to the topic of environmental destruction.

## Honouring the spirit of God in nature

My journey directs me to Jung’s words on the numinous and to Jewish tradition:

A man is made perfect by numen and lumen and by these two alone. Everything springs from these two, and these two are in man, but without them man is nothing, though they can be without man … i.e. of the light of nature … dispersed or sprinkled in and throughout the structure of the great world…. The sparks come from the ‘Ruach Elohim’, the Spirit of God.

(Jung 1947/1954, para. 388)

Jung alludes to the Jewish creation myth of the ‘Shattering of the Vessels’ (Bereshit Rabba 3:7*)*. Here, God contained the divine light within vessels to provide space for humankind to create. The light was powerful and broke the vessels, scattering holy sparks throughout the world. The task of humankind is to discover the sparks and engage in *tikun olam*, repairing the world. This myth emphasizes that every aspect of our Earth contains holiness. The underlying question then becomes, how dare we destroy even a blade a grass? We cannot dismiss anything as secular or mundane, rather it is our responsibility to discover the numinous even within the sand on the beach and the small stone we discover on the path.

## Adam One vs. Adam Two: a Jewish view on environmental destruction

The Jewish philosophical tradition is further related to the topic of stewardship of the environment. Rabbi Joseph B. Soloveitchik (1965/2006) ponders the meaning of two differing accounts of the creation of humankind in the Biblical book of Genesis. Soloveitchik uses them to propose two differing roles for humankind that he designates as Adam One and Adam Two. Creation of Adam One:

So, God created Adam (man) in His own image, in the image of God created He him male and female created He them. And God blessed them, and God said unto them, be fruitful and multiply, and fill the earth and subdue it, and have dominion over the fish of the sea, over the fowl of the heaven, and over the beasts, and all over the earth.

(Soloveitchik 1965/2006, pp. 27‐30)

Creation of Adam Two:

And the eternal God formed Adam (man) of the dust of the ground and breathed into his nostrils the breath of life and man became a living soul. And the eternal God planted a garden eastward in Eden … And the eternal God took the man and placed him in the Garden of Eden to serve it and keep it

(ibid., pp. 7‐15)

Soloveitchik states that the two creation stories highlight two seemingly opposing, yet complementary, roles of human existence. Wo/man first receive the mandate of *imitatio Dei* to subdue and master nature. Adam One asks, ‘How does the world work?’; Adam Two was charged with serving and keeping the garden. In the words of Soloveitchik:

Adam the second is receptive and beholds the world in its original dimensions. He looks for the image of God … in every beam of light, in every bud and blossom, in the morning breeze and the stillness of a starlight evening … Adam the second lives in close union with God.

(Soloveitchik 1965/2006, p. 22)

Rabbi Lord Jonathan Sacks, former chief rabbi of the United Kingdom, carries this paradigm further by commenting on the verb in the second creation story – *leshomarh* – to guard. He ties it to the responsibilities of a guardian of property that belongs to someone else:

This is the verb used in later biblical legislation to describe the responsibilities of a guardian of property that belongs to someone else. This guardian must exercise vigilance while protecting, and is personally liable for losses that occur through negligence. This is perhaps the best short definition of humanity’s responsibility for nature as the Bible conceives it.

We do not own nature – ‘The earth is the Lord’s and the fullness thereof’ (Psalm 24:1). We are its stewards on behalf of God, who created and owns everything. As guardians of the earth, we are duty‐bound to respect its integrity.

(Sacks 2014)

From a Jewish perspective, Soloveitchik and Sacks remind us that the numinous and spiritual must stand side‐by‐side with the worldly. We must be watchful stewards of a world that is not ours.

## The lens of nature and the environment

The second half of this paper focuses on the dreams and sandplay process of three patients and a dream from a dream group participant. In contemplating the topic of this paper, I began to use a more nuanced lens in the temenos of my analytic work. Although dreams have always played a prominent role in my work, my training focused more on the relation of dreams to the personal rather than the collective unconscious. I began to look differently at past dreams that referred to scenes in nature and to the animal world, and I noticed more clearly the impact of the environment and the numinous within the symbolic material of analysis. Where would we be without nature and the environment? We need lions and tigers and bears (Hillman 1997). We gaze into the depths of the sea and we listen for the sounds of thunder. How often does a snake visit us in the night? Imagine if we only dreamed of cities and buildings? Our work of the soul would become dull and dried up like a parched, empty riverbed. Suddenly I felt compelled to uncover the deeper and more objective levels of the psyche, not only in dreams, but also in other symbolic material arising in the consulting room. It is with this magnifying glass that I discovered hints of the objective knowledge of environmental destruction and threats to natural habitats.

An earlier dream of my own needed reconsideration:

I am in my office with a former patient, herself an accomplished and empathic therapist. I am wearing white. The radio is on, although it is the Jewish Shabbat (Sabbath), a time I would neither see patients, nor listen to the radio. I look outside the window and am horrified by what I see. There is a row of tree stumps bereft of their branches, which have been perfectly cut off. What is most disturbing and eerie to me, is that there are absolutely no remnants of this devastation of nature. And I understand an alien force has arrived. I comprehend that my patient and I must quickly escape. Many details follow in which I try to secure forged papers to run away to Iceland. Sadly, I understand I must leave quickly without my family. I associate to the period of the Holocaust.

(Zeiger 2021, p. 607)

This dream, from February 2020 when COVID had not yet arrived in Israel, I understood as an intuitive and numinous dream about COVID. In revisiting the dream, I now wonder at the connection to the objective unconscious. Perhaps there is warning here about what we human beings, as ‘alien forces’, are doing to the environment.

## The environment as powerful background

In the first two dreams, the environment was not the main subject and neither myself nor my patients related to the dreams with concern about the environment. Nevertheless, themes of death and destruction within the natural world emerged from the unconscious.

## Carly and the death of the sea lion

Carly, a woman in her thirties, brought this dream after several months in therapy:

I was under the sea with my boyfriend. He was riding a sea lion. The animal tried to escape. The animal felt tortured. My boyfriend fell to the side. I wanted to catch it and ride it. I sat on the back of the sea lion. I felt it wants to escape from me. It was shaking and moving. It became super‐fast. It was too fast. I needed to release it. I felt anxious. I was inside a huge building. I released it. It hit stone and died. It got smaller and weaker, to a small lizard from a sea lion. Other people surrounded me to ask, ‘Why did you release it inside the building’?

Carly had immigrated to Israel from Europe to develop more independence on her path of individuation. She began therapy complaining of boredom and lack of energy and desired a life‐long partner yet could not decide about her current romantic relationship. Carly was also haunted by superstitious thoughts which had a flavour of death anxiety. These superstitions were perhaps a ritualistic and protective measure for dealing with the ongoing childhood trauma (Kalsched 1996 & 2013) she had suffered related to a punitive stepfather who raised her with fear of sin, punishment and hell. She had developed a strong guilt complex, taking a great deal of responsibility for others, often felt ‘stuck in place’, frightened of change, and struggled with finding a positive attitude to life. She had learned to remain on high alert and suffered with a great deal of death anxiety. It became clear that she had developed what Neumann (1973/2002) refers to as a ‘distress‐ego’ that forced her to be parental while quite young. In Neumann’s words: ‘Where the primal relationship is disturbed, the distress‐ego is prematurely thrown back on itself; it is awakened too soon and driven to independence by the situation of anxiety, hunger and distress’ (1973/2002, p. 77).

## Dream work and the personal unconscious

Dream work for Carly was both new and interesting yet scary for her. Just below the surface sat distrust and fear of the unconscious and death. Meeting the dream with curiosity, the opening scene was characterized by movement, drama and energy. Interestingly, it was the opposite of being stuck. In waking life, Carly longs for a partner to offer answers to her boredom yet in her dream she left her boyfriend ‘fallen’ in the wake of the sea lion ride and continued her journey of individuation alone. While Carly’s dream soul sought the thrill of danger, the ride proved to be too scary, too fast, leaving her shaken and guilty as the sea lion died.

The sea lion as an aspect of nature and the animal kingdom is a powerful and key figure in this dream. At its most obvious, Carly sought energy, libido, and excitement via the drama and danger of the sea lion ride. Her fascination with the sea lion, an animal not meant to be ridden by humans, was a path that led to trouble. The dream seemingly offered a warning against diving too deeply into the depths of the unconscious, perhaps because the dream highlighted her childhood complex of excitement, experienced via drama and danger.

Hillman (1979) reminds us to look beyond the more obvious connections that we often assume; animals are symbolic of instinct and vitality. Rather, we are advised to meet the animal in its own right, ‘as Gods, as divine, as intelligent, autochthonous powers demanding respect’ (p. 147). Further:

To look at them from an underworld perspective means to regard them as carriers of soul, perhaps totem carriers of our own free‐soul or death‐soul, there to help us see in the dark. To find out who they are and what they are doing there in the dream, we must first of all watch the image and pay less attention to our own reactions to it … But no animal ever means one thing only, and no animal simply means death.

(Hilman 1979, p. 148)

Sea lions as carriers of the soul are playful and at home in both water and on land. Carly’s problem was that the ride was unregulated. Unlike our relationships with dolphins, sea lions do not trust humans and can be quite dangerous. As Hillman reminds us, our dream reality is not our waking reality. Perhaps Carly could have ‘made friends’ with the sea lion, perhaps she could have learned from this animal’s skill for entering and exiting the depths of the waters of the unconscious. But the sea lion hit the wall inside the building and died, transforming into a lizard. Here we meet the theme of death and rebirth/transformation into something entirely different. Perhaps the inflated power of Carly and her boyfriend’s ride on the sea lion transformed into something more manageable.

## Environmental destruction via the objective layer of the dream

Carly’s seemingly impulsive and self‐centered journey led to a destruction of the animal. In looking at the dream through the lens of environmental crisis, we can see that the animal ended up trapped inside a building. In consort with my much earlier dream of the lizard, containment in a human structure not meant for the animal world was part of the problem. Often it is humankind’s urbanization without foresight and encroachment on the non‐humanized world that leads to the destruction of natural habitats and the displacement and endangerment of species. The buildings hint at the conflict between urbanization and protection of our natural habitats. Israel is a small, but fast‐growing country. The desert remains relatively undeveloped, yet here too there are environmental warnings of our negative effects on the environment (Kavaler 2020). The desert community that is home to Boomie and my daughter is also home to the Ibex, an endangered species. The co‐existence of humans alongside these majestic creatures is no easy task and the Ibex is now protected by governmental rules.

The personal intersects the archetypal and the theme of this issue. The dream presented the dilemma of modern humankind. Sometimes, as humans with needs, we begin innocently to use something and then come to both abuse and exploit it. A sea lion is not a creature for humans to ride. The masculine force in this dream harnessed and used the unwitting sea lion. Sometimes we can all ‘jump on board’ without sufficient foresight. We destroy something because we can’t see ahead into the darkness. In writing this paper, I sadly discovered that sea lions are on the list of endangered species. 1 I began to wonder how many other dream animals are on the brink of extinction.

## Yaffa & the tsunami of destruction

Perhaps one of the strongest dream images of destruction within nature was brought to me by a bright, creative and energetic young woman I call Yaffa.

I was with my father in a strange place on a lake that was makeshift and topsy‐turvy. It was not solid. There was debris and scaffolding. It looked like a tsunami. He was making up a song and giving it over to me. I was to learn it and I did. Time passed and all of a sudden no one remembered but me.

Yaffa began analysis struggling with depression, anxiety and entangled in a terrible marriage to a man who suffered multiple mental health problems. During analysis, she divorced him and eventually remarried. Dreamt early in the process, this dream served as a guiding beacon for the work, directing us to focus on her father and mother complexes. Yaffa was born into a family with a larger‐than‐life father and a powerful connection to the patriarchal collective. She is from a large religious family. Her deceased father was a popular Chassidic Rebbe (a title of endearment used in place of Rabbi that means ‘my teacher’) with many followers. She admired him greatly and felt lost without his advice.

## Dream work and the personal unconscious

Entering the dream, we meet a scene characterized by contrasts. There is tsunami‐like destruction of Mother Earth paired with scaffolding, something unusual at a lake. The stark contrast suggests the necessity of both destruction and rebuilding. Yaffa stated she was happy to be at the lake alone with her father, who had always been engaged in projects and surrounded by others. Yet, she tells me that she was not happy with the task of remembering her father’s song. She described her own musical talents, and we began to uncover seeds of her own independence hidden behind the voices of others. In the beginning, when I asked Yaffa what she thought, she often told me the opinion of someone else. Much of the work was to discover and strengthen her own voice ‘to sing her own song’.

## Looking through the lens of nature and environment

I found myself returning to this dream in my mind during our two‐year process. I love to sit quietly amongst nature, and thus the dream appeared horrific to me, in the same moment that Yaffa seemed to accept the destruction. She was happy to be alone with her father, yet I was ripe for the strong countertransference that captured me internally at that point. Perhaps, via a form of projective identification, I was meant to become the container of inner destructive rage that ensued from what seemed to me to be the minimization of the destruction of the world of the mother. In revisiting the dream, I see that the father sits at the lake as a symbol of a one‐sided patriarchal ego that ‘protects’ itself and passes on the old song. But Yaffa needs her own song and we need the song of the new way. In revisiting Bernstein’s out of balance Western ego that can commit species suicide, the first step in repair is noticing the wounds and destruction. Something must be rebuilt both in Yaffa’s world and in the world‐at‐large. Yaffa began analysis with a very rational stance. I had extreme difficulty in feeling her in the room. On the surface, her intellectual capabilities and verbal abilities suggested a well‐developed ego, yet it quickly became clear that she too had developed a distress ego. She was overly responsible and attentive to the needs of others, mothering her own mother.

The song is an important symbol in the archetypal story. Yaffa did not seem to have access to her mother’s song. We often associate night‐time lullabies with this world of mothering, and as Neumann (1973/2002) points out, a good primary relationship helps with sleep. Yaffa told me about her sleep disturbances, as well as sad stories about how, as a child, she put herself to bed. The missing mother’s song is yet another image from the collective unconscious of a world unbalanced. Of note, it was the loss of her powerful father that propelled Yaffa to embark on the path to her own song.

## Faith and ‘Majesty’

Faith sought out therapy in her mid‐thirties to deal with relationship issues. Bright, highly educated, and creative, she had earlier immigrated from the U.S. but because of difficulties with the Hebrew language, she gave up work in the arts and mental health to work in various technology start‐up companies. A staunch vegan and fierce protector of animal and human rights, she attempts to protect the underprivileged (whether neglected humans or animals), sometimes at her own emotional and financial expense. Born to a single mother with a history of substance abuse and undiagnosed mental illness, Faith was seriously neglected as a child and was forced to grow up too quickly. Although she suffered many traumatic childhood experiences, e.g. foster homes, abusive adoptive parents, childhood rape by a half‐sibling, incarceration of her mother for drug involvement, 2 there were several aspects of her story that helped save Faith from more serious problems, most notably her companion ‘Majesty’.

## The ‘sandtray’ and the personal unconscious

Of particular importance to this work is Faith’s deep connection to nature. Mother Nature can sometimes compensate for maternal neglect and substitute for or supplement poor mothering (Kalsched 1996, 2013). From an early age, Faith loved nature and became connected to dogs, cats, wolves, and other animals. There were times she lived as a hippie in a van, accompanied by her half‐dog/half‐wolf whom I will call Majesty. It was if she was more at home in nature, amongst the trees and creatures, than she was in the city. Bernstein (2005) refers to people such as Faith as ‘borderland’. They are highly connected to and impacted by nature:

Borderland people personally experience, and must live out, the split from nature on which the ﻿western ego, as we know it, has been built. They feel (not feel about) the extinction of species; they feel (not feel about) the plight of animals that are no longer permitted to live by their own instincts, and which survive only in domesticated states to be used as pets or food.

(Bernstein 2005, p. 9)

They are deeply feeling, sometimes to such a degree that they find themselves in profound feeling states that seem irrational to them. Virtually all of them are highly sensitive on a bodily level. They experience the rape of the land in their bodies, they ﻿psychically, and sometimes physically, gasp at the poisoning of the atmosphere … This psychic identity with the animate and inanimate objects of nature is a phenomenon that anthropologist Lucien Levy‐Bruhl recognized among native cultures, and which he called *participation mystique*.

(ibid., p. 9)

The deep extent of Faith’s trauma emerged via our relationship. She reported horrific stories without any emotion. She displayed poor boundaries that allowed her to dangerously live on the edge. We began to meet the memories and feelings via the non‐verbal, i.e. through art and sandplay. This was crucial to her process of recovering some of her buried emotions.

**Figure 1:**
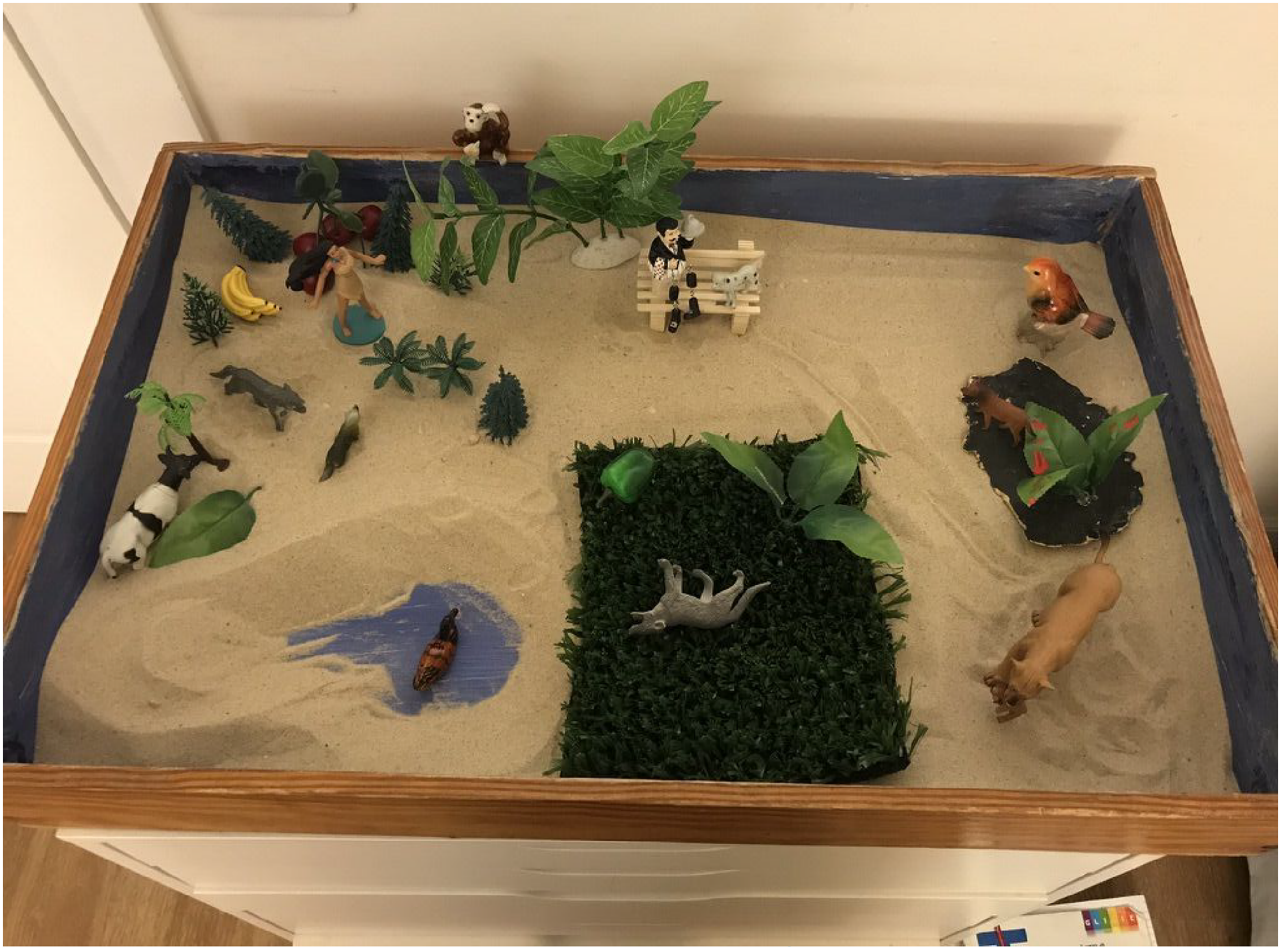
The importance of nature in Faith's inner world

Faith created this sandplay scene (Figure 1) three years into our process and about four months after Majesty died. The loss of Majesty as soul‐animal and guardian was perhaps Faith’s most devastating loss. Faith created a miniature Majesty of clay and used it in her sandplay work. She kept her in a coffin‐like box in my office in between sessions. I find it interesting to reflect upon the fact that I felt the need to change Faith’s pet’s real name to protect confidentiality. This is the first time that I have done this.

On the day of this sandplay session, Faith arrived tired, angry, and burned out from an unenjoyable job saying she wanted to ‘just be’. Slowly, in a meditative stance, Faith built a colourful and nature filled scene with a Native American woman, trees, water, grass, and a collection of domestic and wild animals (e.g. dogs, duck, bird perched, cow, wolf, and Majesty on the grass). She placed a ‘poor man with broken legs’ on a bench. A monkey with bananas sat on the side of the tray. Faith also added a lioness holding her cub in her mouth, a familiar figure that she used and discussed many times. Faith stated:

The tray is a happy world of feeding dogs. My character is Native American. This is my ego‐self/Inner self. She is shoeless. I like him [mountain lion]. Would camp out in Utah. People worried about mountain lions. We had to sleep with campfires. For me, he was watchful, wary. We both had our boundaries. [Faith then refers to her frequent dreams of mountain lions over the years.] In the dream, there is often a little path. He is hiking. He looks at me and then turns his backside to me. Then he keeps going. He gives me permission to go. I have an understanding with him. He is dangerous. He is a teacher of boundaries.

Faith’s tray spoke of her deep connection to nature and to animals to soothe and inspire her. Her tray allowed me to ‘enter her world’ and witness first‐hand both her trauma, followed by the reparative force of nature. Sandplay is a technique that allows the builder to create a piece of their inner world (De Domenico 1988). The therapist begins as a witness and observer and with the patient’s invitation, s/he symbolically enters the patient’s world.

In Faith’s words, this tray was a happy tray. Yet there were hints of the trauma and loss (a man with broken legs, memories of dangerous mountain lions, and the mourning of her beloved four‐legged companion). Faith identified with the Native American woman who is at home in this world of nature. Both the Native American woman and the lion appear to be something more than a symbol for Faith. They served also as concrete testimony to her deep connection to nature as both protector and guide. The mountain lion taught her about boundaries, something that was never to be easy for her. It was interesting that Faith referred to the lioness carrying the cub as ‘he’. It was startling enough for me that I looked online to discover if males carry their cubs. Perhaps she unconsciously replaced her ineffective mother and abandonment by her father, with an image of a protective and fierce great father.

Interestingly, Faith had chosen leaves covered in red paint. She had no conscious idea, but I had created this piece previously as a symbol of pain. The red was a reference to blood. Perhaps this piece conveys the feeling tone of the tray. From a distance, the tray looked colourful and ‘nice’, filled with natural objects from the monkey to the flowers to the bananas. Yet, the blood of suffering permeated the underbelly of this tray. Kalsched’s (1996, 2013) model of trauma is useful in understanding Faith’s journey and suffering. She grew up relying upon the assistance of Mother Nature to compensate for the neglect. In her glory, awe, and fear, the Great and Terrible Mother provided protection, warning and boundaries. Faith’s connection to animals and nature offered her soothing, as well as lessons of life. Majesty and all the wounded animals Faith rescued over the years became her children, offering a symbolic way to tend to her wounded child.

For Faith, the Native‐American woman was treasured. While Faith had no contact with her biological father, she mentioned during our process that her mother thought the father had Native‐American blood. Faith became very interested in Native American peyote ceremonies and lived very close to an Indian reservation as a young adult. Perhaps most importantly, in consort with Bernstein, Faith had always experienced viscerally the plight of nature and of animals. It has appeared much stronger than empathy, as if she has always been in *participation mystique* with our environment. As Bernstein posits, some people are born into a borderland existence. Perhaps this is Faith, or perhaps her trauma prompted her to adopt Mother Nature as her mother. It may also have something to do with her Native‐American heritage.

## Looking through the lens of the objective psyche

To my mind, it is not an accident that Faith became a citizen of the world. Her substitute mother was to be found in deep connection with nature, and thus, in like kind, she offers this nurturance to the world at large. I was truly honoured to have accompanied Faith on her journey and was inspired by her resilience and compassion for the world. Bernstein points out how the borderland individual offers a heroic and deeply important stance for our world.

When I have the opportunity of working with Borderland personalities, I am moved not only by their struggle to do their personal work, but to do our work for us as well, that is, for those of us who are much less connected to and in touch with the Borderland. I see the deeper thrust of this new phase in our psychic evolution as a pulling back from the brink of self‐extinction. It is in this sense that Borderland people are the unrecognized heroes and heroines of our collective evolution toward growth, consciousness, and individuation. Theirs is a large and sacred work. To the extent this is true, we all will stand or fall with the outcome.

(Bernstein 2005, p. 13)

Thus, in summary, Bernstein points out the necessity of individuals like Faith who are engaged in sacred work. I chose my patient’s pseudonym before I read Bernstein. Perhaps it is no accident that I associate her with faith and hope.

## The birth of an ant

The final dream was brought to a dream workshop I organized in the same desert community in which Boomie and my daughter live. The dreamer, who I will call Tal, was studying at the University of Desert Studies at the time of the dream.

I dreamt I was in a village in India that was very colourful. There was a festival in the background. I see C, a German woman I found on a trip in a forest in Momar, a city in southern India. She gave me a good deal of confidence how to learn about plants. C encourages me and helps me with the contractions and pain of birth. Then, I see the guy that I slept with, and there are no interactions between us other than he says, ‘Hi, how are you?’. I find a friend who is a psychologist I met when studying for my bachelors. I meet other people. This is strange because they are with me in the experience of birth. They are simply there walking around. I give birth to an ant that is very beautiful. She is brownish red with a bit of black and big. She is in danger of extinction. Because of this C encourages me and says to me that I am doing something good and very important for the population of ants. The birth ends and the festival in the village continues. We remain there and there is around us a circle of people that are supportive but not close to me. A person who is standing close to me is a male dancer from the festival.

Tal was studying desert ants that are restricted to a specific habitat where the soil is unique and there are not many rocky outcrops. In Tal’s words, ‘I do feel I appreciate ants a lot because of how they navigate and function as a dispersal agent for rare plants’. Tal felt that the dancer represented a symbol of freedom of movement. In her words:

‘The fact that I do not remember any shocking faces in the dream, means that I was in a very comfortable environment where I feel happy and ‘at home’. I am making a lot of effort to keep myself surrounded with conscious people who care about climate change. I often take it personally when people are not taking it seriously. I think that the dream made me appreciate my closed environment and the friends I am surrounded with’.

Tal’s professional stance as researcher/observer is in contrast to her experience of the dream. The opening scene is framed within the power of the feminine: Tal is pregnant and giving birth to an ant that is beautiful but is in danger of extinction. The actual man who had impregnated Tal appeared as an insignificant character, suggesting an ‘impersonal’ or archetypal impregnation. The birth hints at transformation within Tal. She was assisted in her labour by a powerful female figure who she had previously met in India, a place of journey and often spiritual awakening. This woman was knowledgeable about the plant world and she took on a midwifery role. Perhaps here is the development of an inner midwife of transformation for Tal in her research work with ants. Interestingly, Tal’s ants are dispersal agents for the seeds of rare plants and, in contrast to the almost anonymous donation of male sperm, these seeds play a vital role in protecting rare species from extinction.

The background in the dream was colourful, dynamic, and filled with supportive onlookers. Tal met someone from the world of psychology, hinting at inner development, a male dancer, perhaps symbolic of integration between the masculine and the feminine. Finally, in Tal’s words, she was touched by the supportive people who surrounded her, while respecting her need to birth herself.

I am grateful for the creative and novel productions of the unconscious. In my almost four decades of work as a psychologist, I have never heard of someone birthing an ant. Tal could not have consciously known of my plan to write this paper on climate change, yet it felt as if her unconscious contributed an incredible gift for the end of the paper. Relating to my request to include the dream, she spoke of her deep commitment to the environment and her appreciation of community.

Tal’s dream hinted at her own path of individuation as a researcher. Like Boomie, perhaps Tal and her midwife served as bridges between the masculine, often ego‐possessed scientist and Mother Earth. We need science, logos, and a strong ego function to progress. Yet, these forces must be held and contained within the wisdom of the feminine. What do ants symbolize? Ants are hard workers. They live in colonies. We project onto them a strong work ethic within a tight collective. They are extremely small and often go unnoticed, unless they bother our human existence by daring to enter our abode. Then it is all too easy to stomp on them, curse them, and poison them. Children are often fascinated to watch ants march along, until one day, they too lose interest and become equally annoyed with their intrusiveness. How easy it would be with our hubris to exterminate all the ants in the world?

With these reflections on the importance of the tiny ants to enrich our world, I am brought back to Vaughan’s charge to become humble. In the brief moments in which I was privileged to meet and connect with Tal, I was impressed and humbled by her work to make the world a better place.

## Afterthought

I return now to my initial conversations with experienced analysts and therapists who responded that they too do not ‘hear’ about environmental destruction in the treatment room. Perhaps many of us are initiates in this area. While revising this manuscript, I had several experiences of synchronicity. As I write these words, an article appeared in the *New York Times*, ‘Climate Change Enters the Therapy Room’ (Barry, Feb. 6, 2022). As I have begun to think more of the topic, I now hear patients speak about climate destruction. I believe that the transformation within me somehow unconsciously invites the transformation in my patients. One woman spontaneously spoke of a horrific experience watching the movie, ‘Don’t Look Up’, in which a comet destroys Earth due to humankind’s greed. Another young woman spoke about her childhood fears of a meteor destroying the world and/or the sun exploding. A sensitive and bright young borderland man, who is now at the beginning of his process, spoke about how ‘horribly, deeply sad it is that people are living in disharmony with nature’. He recently brought me a powerful dream in which he is a deer in the forest. It is no accident. Our souls deeply connect to one another via *anima mundi*. The alchemical transformation in the room is a process between two individuals, both wounded souls and wounded healers.

In closing, I want to share a recent, powerful experience. I participated in an experiential retreat with members of my analytic institute in Israel. Two by two, we entered and meditated in a small cave connected to an important mythological story from the second century of Rabbi Shimon bar Yochai. As a famous Jewish sage, he retreated with his son to this cave during a tumultuous time in Jewish history. Legend states that they were protected and fed food and Jewish knowledge by miraculous Divine intervention. Twelve years later, when they emerged from the cave, the rabbi could not deal with the secular world of day‐to‐day existence and used his eyes to burn up both people and the natural world. The Divine Creator sent the two back to the cave for another year, and this time, they emerged capable of inhabiting the real world. The discussion at the retreat focused on our tendency as depth‐psychologists to remain in the comfortable cave of introspection. Yet, as we discussed outer problems, such as war, terror, and refugees, we reiterated the necessity to live a balanced life of ‘inside and outside’. If we do not look and see with the eyes of our soul, we may burn up and truly destroy our world. My paper ends here, but my journey continues enriched by my patients, my dream world, my colleagues, and my fellow human beings of this world we must protect. We must enter the dark to preserve the light in the world.

## Footnotes

1
http://endangeredspeciesearth.com/sea‐lions.html (last accessed April 21, 2021).

2 An exploration of these issues is beyond the scope of this paper.
